# Longitudinal Trends in Severe Dyslipidemia in the Czech Population: The Czech MONICA and Czech Post-MONICA Study

**DOI:** 10.3390/jcdd10080328

**Published:** 2023-07-31

**Authors:** Renata Cífková, Jan Bruthans, Peter Wohlfahrt, Alena Hrubeš Krajčoviechová, Pavel Šulc, Marie Jozífová, Lenka Eremiášová, Jan Pudil, Aleš Linhart, Jiří Widimský, Jan Filipovský, Otto Mayer, Rudolf Poledne, Petr Stávek, Věra Lánská, Larysa Strilchuk

**Affiliations:** 1 Center for Cardiovascular Prevention, Charles University in Prague, First Faculty of Medicine and Thomayer University Hospital, 140 59 Prague, Czech Republic; jan.bruthans@ftn.cz (J.B.); wohlp@gmail.com (P.W.); alena.krajcoviechova@gmail.com (A.H.K.); pavel.sulc@yahoo.com (P.Š.); marie.jozifova@ftn.cz (M.J.); larysa.stril4uk@ukr.net (L.S.); 2 Department of Medicine II, Charles University in Prague, First Faculty of Medicine, 128 08 Prague, Czech Republic; lenka.eremiasova@vfn.cz (L.E.); jan.pudil@vfn.cz (J.P.); alinh@lf1.cuni.cz (A.L.); 3 Department of Medicine III, Charles University in Prague, First Faculty of Medicine, 128 08 Prague, Czech Republic; jwidi@lf1.cuni.cz; 4 Department of Medicine II, Faculty of Medicine, Charles University, 301 00 Pilsen, Czech Republic; filipovsky@fnplzen.cz (J.F.);; 5 Atherosclerosis Research Laboratory, Institute for Clinical and Experimental Medicine, 140 21 Prague, Czech Republicpetr.stavek@ikem.cz (P.S.); 6 Medical Statistics Unit, Institute for Clinical and Experimental Medicine, 140 21 Prague, Czech Republic; vera.lanska@ikem.cz; 7 Department of Therapy №1, Medical Diagnostics, Hematology and Transfusiology, Lviv Danylo Halytsky National Medical University, 79010 Lviv, Ukraine

**Keywords:** prevalence of severe dyslipidemia, cross-sectional survey, population random sample, total cholesterol, LDL cholesterol, HDL cholesterol, familial hypercholesterolemia

## Abstract

**Background:** Severe hypercholesterolemia is associated with an increase in the risk of developing atherosclerotic cardiovascular disease. The aim of this analysis was to assess longitudinal trends in severe dyslipidemia (defined as total cholesterol > 8 mmol/L or LDL-cholesterol > 5 mmol/L) in a representative population sample of the Czech Republic and to analyze the longitudinal trends in the basic characteristics of individuals with severe dyslipidemia. **Methods:** Seven independent cross-sectional surveys were organized in the Czech Republic to screen for major cardiovascular risk factors (from 1985 to 2015–2018). A total of 20,443 randomly selected individuals aged 25–64 years were examined. **Results:** The overall prevalence of severe dyslipidemia was 6.6%, with a significant downward trend from the fifth survey onwards (2000/2001). Over the study period of 30+ years, the individuals with severe dyslipidemia became older, increased in BMI, and did not change their smoking habits. Total cholesterol and non-HDL-cholesterol decreased significantly in both sexes throughout the duration of the study. **Conclusions:** Despite a significant improvement in lipids in the Czech Republic from 1985, substantially contributing to the decline in cardiovascular mortality, the number of individuals with severe dyslipidemia remained high, and in most cases, they were newly detected during our screening examinations and were thus untreated.

## 1. Introduction

Cardiovascular diseases (CVD) are the main cause of death in most countries worldwide, including the Czech Republic, currently contributing 39.7% of total mortality in men and 44.0% in women. A decline in cardiovascular mortality has been documented in most developed countries over the last 30–40 years [1]. Central and Eastern European countries have also experienced this decline, though substantially later. 

Since 1985, a decline in CVD mortality in the Czech Republic has been noted in both sexes [2]. However, according to the European Society of Cardiology classification, the Czech Republic is still labeled as a country with high CVD mortality [3]. The 2019 ESC/EAS Guidelines state that increased LDL cholesterol values are causally related to atherosclerotic CVD, and that lowering LDL particles and other ApoB-containing lipoproteins reduces cardiovascular events [4]. Using the validated IMPACT mortality model, we showed that the decline in coronary heart disease (CHD) mortality in the Czech Republic between 1985 and 2007 can mostly be explained by the improvement in major cardiovascular risk factors, with total cholesterol contributing 39.5% of the CHD mortality reduction [5]. A significant decline in total and non-HDL cholesterol was documented in both sexes in the Czech Republic and was largely induced by changes in diet [6].

Individuals with severe dyslipidemia have a five-times-higher risk of coronary heart disease and CVD of atherosclerotic origin [7]. Early identification and adequate management, including lifestyle changes and drug treatment, may significantly improve their prognosis and thus reduce the economic burden of CVD worldwide. Severe dyslipidemia in the Czech population has never been assessed before.

As there is a knowledge gap regarding the prevalence of severe dyslipidemia, the aim of this analysis was to assess longitudinal trends in severe dyslipidemia, defined as total cholesterol > 8 mmol/L or LDL cholesterol > 5 mmol/L, in a representative population sample of the Czech Republic from 1985 to 2015–2018. Another aim was to analyze the longitudinal trends of the basic characteristics of individuals with severe dyslipidemia.

## 2. Materials and Methods

### 2.1. Study Population

Altogether, there were seven cross-sectional independent surveys organized to screen for major CV risk factors in the Czech Republic. The first three surveys (1985, 1988, and 1992) were conducted in six districts of the country as part of the WHO MONICA Project (Czech MONICA) [8], whereas the following four surveys (1997/1998, 2000/2001, 2007/2008, and 2015–2018) comprised nine districts, known as the Czech Post-MONICA Study. 

The study population, aged 25–64 years, were always randomly selected as a 1% population sample from each district, stratified by age, sex, and community size. The selection was performed either from the national population registry (first three surveys) or from the General Health Insurance Company registry (subsequent three surveys) and from the registers of five major health insurance companies operating in the Czech Republic, which covered 85% of the total population (for the most recent survey). The details are published elsewhere [6,9], but it is important to note that health insurance is mandatory in the Czech Republic and is paid for by the employer/employee or by the government for children, the retired, and the unemployed. 

The Ethics Committee of the Institute for Clinical and Experimental Medicine and Thomayer University Hospital, Prague, Czech Republic, approved the Czech MONICA and Czech Post-MONICA Studies. Informed consent was obtained from all the participants.

### 2.2. Screening Examination

The examination consisted of a questionnaire, which was completed by the examining physician. Starting from the fourth survey, the questionnaire was extended to include information about any history of CVD and currently prescribed medications, which were verified against their containers when possible.

Height and body weight measurements were taken while standing, without shoes and outer garments. Blood pressure (BP) was measured consistently on the right arm (properly supported at the heart level) while seated, after a minimum five minutes of rest, employing standard mercury sphygmomanometers using the correct cuff size. All BP values were recorded to the nearest 2 mmHg. In the first three surveys, only two BP measurements were taken, whereas for the last four surveys, three consecutive BP measurements were performed. For the analysis of longitudinal trends, only the mean of the first two readings was used.

A venous blood sample was taken gently while the individual was seated after fasting for a minimum of 12 h. The blood samples were centrifuged at 1500 G and frozen immediately thereafter.

### 2.3. Laboratory Analysis

The lipid parameters from all seven surveys were analyzed in the same Lipid Laboratory of the Institute for Clinical and Experimental Medicine, Prague, Czech Republic, which served as the WHO Reference Laboratory for the duration of the WHO MONICA Project.

The MONICA Manual provides precise rules regarding the standardization of the analysis of lipid parameters. In the first three surveys, only total cholesterol (determined with an enzymatic method using CHOD-PAP kits, Boehringer, Mannheim, Germany) and HDL cholesterol were analyzed (also assessed using an enzymatic method after the precipitation of serum apolipoprotein B-containing lipoproteins with sodium phosphotungstate). LDL cholesterol was calculated using the Friedewald formula [10] if the triglycerides were ≤4.5 mmol/L. From the fourth cross-sectional survey in 1997/1998, the lipid parameters were determined using a fully automated enzymatic method (COBAS MIRA S analyzer, Roche Diagnostics, Indianapolis, IN, USA) with enzymatic kits produced by the manufacturer. 

The Centers for Disease Control and Prevention (Atlanta, GA, USA) monitored and tested all the analyses for accuracy. Total cholesterol and HDL-cholesterol were found to be within the limit of ±2%.

Glycemia was determined using an enzymatic method (kits from Lachema, Brno, Czech Republic). 

### 2.4. Definition of Major Risk Factors

Current smokers were defined as smoking at least one cigarette per day for the WHO MONICA Project [11]. Hypertension was defined as a mean systolic blood pressure (SBP) ≥ 140 mmHg and/or a mean diastolic blood pressure (DBP) ≥ 90 mmHg or current treatment with antihypertensive drugs [2]. Individuals were labeled as having diabetes if their fasting glycemia was ≥7 mmol/L or if they were treated with oral glucose-lowering drugs or insulin [12]. We defined severe dyslipidemia as total cholesterol > 8 mmol/L or LDL cholesterol > 5 mmol/L. 

### 2.5. Statistical Analysis

Statistical analyses were performed using JMP^®^ 16.2.0 statistical software (2020–2021, SAS Institute Inc., Cary, NC, USA). Trends for the means were tested based on linear contrast in a one-way ANOVA, and for triglycerides, logarithmic transformation was applied. Trends for percentages were analyzed using the Cochran–Armitage trend test for nominal variables and linear regression for ordinal ones, and then Bonferroni correction was applied for the adjustment of the *p* values. A two-sided *p* value ˂ 0.05 was considered significant.

## 3. Results

### 3.1. Characteristics of Population Samples 

A total number of 20,443 individuals of European descent were examined in seven independent cross-sectional surveys (Table 1). Women showed a consistently slightly higher response rate. A significant downward linear trend was seen in the response rates of both sexes, with a particularly sharp decline for the last survey, especially in the youngest age group. 

### 3.2. Prevalence of Severe Dyslipidemia

**Figure 1:**
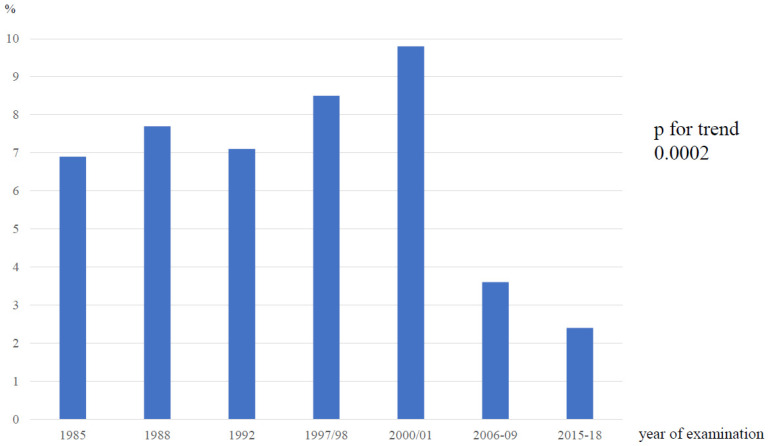
Prevalence of severe dyslipidemia, Czech Republic, from 1985 to 2015–2018.

The overall prevalence of severe dyslipidemia in our population between 1985 and 2015–2018 was 6.6% (1351 cases out of 20,443 screened individuals), varying between 2.4 and 9.8% (Figure 1), with a significant downward trend from the fifth survey (*p* for trend 0.0002).

### 3.3. Longitudinal Trends in Characteristics of Individuals with Severe Dyslipidemia

**Figure 2:**
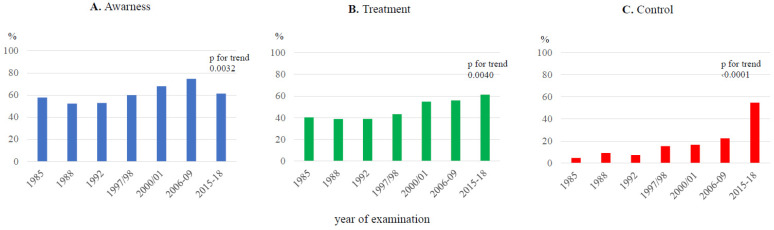
Awareness (**A**), treatment (**B**), and control (**C**) of concomitant hypertension in severe dyslipidemia, Czech Republic, from 1985 to 2015–2018.

There was a significant increase in the mean age of individuals with severe dyslipidemia over the duration of the seven cross-sectional surveys (Table 2). The same trend was observed for BMI (1985: 28.6 ± 4.52 kg/m^2^; 2015–2018: 29.6 ± 4.18 kg/m^2^; *p* = 0.039). Unfortunately, the prevalence of smoking among individuals with severe dyslipidemia remained unchanged over the study period, varying from 27.5% to 37.6%. There was a significant decrease in BP (both SBP and DBP), resulting in a decline in the prevalence of hypertension. Figure 2 shows the awareness, treatment, and control of hypertension among individuals with severe dyslipidemia, indicating a significant improvement in all these parameters over the duration of the study. Data on the prevalence of diabetes were available only from the fourth survey onwards, showing no increase and varying between 4.7 and 10%. Similarly, data on self-reported cardiovascular disease of atherosclerotic origin (ASCVD) were also available only from the fourth survey, with a significant decline in its prevalence from 1997/1998 to 2015–2018.

### 3.4. Longitudinal Trends in Lipid Parameters of Individuals with Severe Dyslipidemia

Regarding the lipid parameters, total cholesterol and non-HDL-cholesterol decreased significantly during the study period in both sexes (Table 3), whereas triglycerides and LDL-cholesterol only improved in women. Notably, data on triglycerides, calculated LDL-cholesterol, and the use of lipid-lowering drugs were available only for the last four surveys. The use of lipid-lowering drugs was low in both sexes and showed no change over time. 

## 4. Discussion

In a representative middle-aged Czech population, we found the prevalence of severe dyslipidemia to be 6.6%, with a downward trend from the beginning of the present millennium. The overwhelming majority of individuals with severe dyslipidemia were newly detected and therefore not treated. This is an alarming finding, as severe hypercholesterolemia is associated with a substantial increase in the risk of developing CVD of atherosclerotic origin [7]. Early diagnosis and aggressive treatment significantly reduce the burden of CVD [13].

Our prevalence of severe dyslipidemia is comparable with that observed in other studies, including the National Health and Nutrition Examination Survey (NHANES), which analyzed data from 1999 to 2014 among adults aged 20 years and older and found severe dyslipidemia (LDL cholesterol ≥ 190 mg/dL~4.91 mmol/L) to be 6.6% [14]. 

A retrospective, record-based, cross-sectional study in Kentucky, USA, found the overall prevalence of primary severe hypercholesterolemia, defined as LDL cholesterol ≥ 190 mg/dL (4.91 mmol/L), to be 7.4% during the period between 2009 and 2020, extracting data from 289,299 medical records [14]. A similar methodology using the electronic health records of primary care settings was employed in Olmstead County, Minnesota, USA, finding the point prevalence of severe hypercholesterolemia to be 4.44% [15].

Reports on severe dyslipidemia in Central and Eastern Europe are scarce. The Lithuanian High Cardiovascular Risk Primary Prevention Program for the identification of patients at high risk of CVD found high LDL cholesterol, defined as ≥6 mmol/L, in 3.4% of their study group, comprised of men aged 40–55 years and women aged 50–65 years without overt CVD. If severe dyslipidemia was extensively defined as increased total cholesterol ≥ 7.5 mmol/L, increased LDL cholesterol ≥ 6 mmol/L, or triglycerides ≥ 4.5 mmol/L, the prevalence increased to 13.5% [16]. 

An alarming finding of our study is that approximately one third of individuals with severe dyslipidemia are current smokers, with no significant change in their habits appearing over time. There is no doubt that the risk of CVD in individuals with severe dyslipidemia is particularly enhanced by smoking [17].

In our population of individuals with severe dyslipidemia, the longitudinal trends in the remaining risk factors, such as BMI, BP, and hypertension prevalence, mirror the trends in the entire examined population [6].

Another striking issue is the fact that 48.4–60.6% of individuals with severe dyslipidemia also had hypertension, which had previously been detected in most of them (52.8–67.9%). The proportion of cases with severe dyslipidemia treated with antihypertensive drugs increased over time. Unfortunately, these individuals continued to have poor control of their hypertension, which was worse than that in the general population [6]. The current hypertension guidelines clearly recommend providing statins to hypertensive patients without previous CVD and those at moderate to high CV risk if their LDL cholesterol value is above 3 mmol/L [18]. Therefore, a substantial proportion of individuals with severe dyslipidemia could have been identified through a proper work-up of their hypertension.

The use of lipid-lowering drugs in individuals with severe dyslipidemia was found to be less than that in the general population in the Czech Republic [6]. This is very alarming, as half of the cases with severe dyslipidemia had concomitant hypertension, clearly increasing their total cardiovascular risk. 

### 4.1. Strengths and Limitations

A major strength of our study is that the results were obtained from representative population samples of the Czech Republic, accounting for seasonal variations. The methods employed were standardized, having originally been designed for the WHO MONICA Project [19]. It is important to note that all the lipid parameters were assessed in the same lipid laboratory, which served as the reference laboratory for the WHO MONICA Project and was monitored by the Centers for Disease Control and Prevention (Atlanta, GA, USA). The BP measurement technique remained constant throughout all seven cross-sectional surveys, using mercury sphygmomanometers, which are still considered the gold standard. Interestingly, the 32-year study period witnessed a regime change from totalitarian to democratic, associated with major socioeconomic changes and their consequences, both positive and negative.

A possible limitation of our study is the decreasing response rate, potentially resulting in a population sample with slightly different socioeconomic parameters, such as higher education. Highly educated individuals are more health-conscious in general. Therefore, the results may be better than the actual situation.

It is estimated that among individuals with severe dyslipidemia, approximately 5% have clinically defined familial hypercholesterolemia (FH), an autosomal dominant monogenic disorder, for which a definitive diagnosis can be made via genetic screening [7]. However, mutations are currently only detected in 30–50% of patients with a clinical diagnosis of FH [20]. Among 20,485 controls free of CHD and prospective cohort participants, there were 6.7% with severe hypercholesterolemia, defined as LDL cholesterol ≥ 190 mg/dL (4.91 mmol/L). Of these, only 1.7% were found to have an FH mutation [21]. Because genetic testing is not available for the majority of treating physicians, including specialized clinics, a simplified definition was proposed in Canada. Probable FH is now simply defined based on the age-specific 95th percentile for LDL cholesterol and a diagnosis of premature CVD of atherosclerotic origin for the patient or a first-degree relative [22].

### 4.2. Implications for the General Population

As our results were obtained from representative population samples, the results could be extrapolated to the entire population of the Czech Republic. The age range of our study group of 25–64 years currently accounts for approximately 50% of the entire country’s population of 10.5 million. Considering the prevalence of severe dyslipidemia of 2.2% obtained during the last screening period, this means that we have roughly 100,000 individuals with severe dyslipidemia who are at high CV risk. 

## 5. Conclusions

The overall prevalence of severe dyslipidemia in seven representative cross-sectional surveys in the Czech population from 1985 to 2015–2018 was 6.6%. Despite a significant decline over time, the number of individuals with severe dyslipidemia remained high, with most of them being newly detected and therefore untreated. Concomitant hypertension was diagnosed in more than 50% of these cases, thus indicating a missed opportunity for initiating adequate treatment.

## Figures and Tables

**Table 1:** Survey sample sizes and response rates.

	1985	1988	1992	1997/98	2000/01	2006–2009	2015–2018	*p* for trend
Total	2570	2768	2343	3208	3320	3612	2621	
Mean age, years	44.9 ± 11.4	45.1 ± 11.3	44.7 ± 10.9	45.6 ± 10.6	46.4 ± 11.0	47.1 ± 11.3	48.0 ± 10.9	<0.001
Men	1253	1357	1134	1538	1627	1737	1250	
Mean age, years	45.0 ± 11.4	45.3 ± 11.3	44.6 ± 10.8	45.6 ± 10.7	46.6± 11.0	47.8 ± 11.5	48.3 ± 10.9	<0.001
Response rate (%)	81.5	85.5	73.2	62.5	63.8	62.1	43.1	<0.001
Age group, *n* (%)								
25–34	307 (24.5)	322 (23.7)	246 (21.7)	317 (20.6)	301 (18.5)	316 (18.2)	185 (14.8)	<0.001
35–44	296 (23.6)	323 (23.8)	350 (30.9)	372 (24.2)	390 (24.0)	401 (23.1)	300 (24.0)	ns
45–54	334 (26.7)	361 (26.6)	310 (27.3)	507 (33.0)	472 (29.0)	404 (23.3)	332 (26.6)	ns
55–64	316 (25.2)	351 (25.9)	228 (20.1)	342 (23.2)	464 (28.5)	616 (35.5)	433 (34.6)	ns
Women	1317	1411	1209	1670	1693	1875	1371	
Mean age, years	44.9 ± 11.4	44.9 ± 11.2	44.9 ± 11.0	45.5 ± 10.6	46.2 ± 11.1	46.5 ± 11.2	47.7 ± 11.0	<0.001
Response rate (%)	85.0	88.4	76.7	66.2	64.8	63.1	48.6	<0.001
Age group, *n* (%)								
25–34	322 (24.4)	342 (24.2)	266 (22.0)	331 (19.8)	330 (19.5)	377 (20.1)	224 (16.3)	<0.01
35–44	340 (25.8)	369 (26.2)	356 (29.4)	446 (26.7)	434 (25.6)	461 (24.6)	312 (22.8)	ns
45–54	343 (26.0)	360 (25.5)	311 (25.7)	532 (31.9)	465 (27.5)	502 (26.8)	420 (30.6)	ns
55–64	312 (23.7)	340 (24.1)	276 (22.8)	361 (21.6)	464 (27.4)	535 (28.5)	415 (30.3)	ns

Data are presented as means ± standard deviation or frequency (percentage); ns—non significant, indicating a *p* value > 0.1.

**Table 2:** Characteristics of individuals with severe dyslipidemia, Czech Republic, from 1985 to 2015–2018.

	1985	1988	1992	1997/98	2000/01	2006–2009	2015–2018	*p* for trend
Number	178	213	167	273	326	130	64	
Age, years	49.9 ± 10.6	51.0 ± 9.4	51.9 ± 8.8	51.0 ± 8.8	51.9 ± 9.3	52.7 ± 9.1	53.1 ± 8.1	0.005
M/F	83/95	102/111	80/87	126/147	152/174	69/61	38/26	ns
BMI, kg/m^2^	28.6 ± 4.52	28.6 ± 4.39	28.7 ± 4.21	28.7 ± 4.24	29.6 ± 4.97	29.1 ± 4.82	29.6 ± 4.18	0.039
Current smoking (%)	67 (37.6)	74 (34.7)	46 (27.5)	93 (34.1)	106 (32.5)	40 (30.8)	21 (32.8)	ns
SBP, mmHg	139.8 ± 20.7	138.2 ± 20.1	140.6 ± 22.4	133.9 ± 17.5	134.0 ± 18.7	136.7 ± 19.6	131.8 ± 16.0	0.002
DBP, mm Hg	86.7 ± 11.4	85.2 ± 10.7	88.3 ± 12.3	85.5 ± 10.0	84.4 ± 10.3	84.5 ± 11.0	84.7 ± 10.6	0.014
Hypertension (%)	104 (58.4)	129 (60.6)	108 (64.7)	143 (52.4)	168 (51.5)	75 (57.7)	31 (48.4)	0.023
Diabetes (%)	-	-	-	26 (9.5)	21 (6.4)	13 (10.0)	3 (4.7)	ns
Personal history of ASCVD (%)	-	-	-	26 (9.5)	19 (5.8)	4 (3.1)	1 (1.6)	0.002

ASCVD—cardiovascular disease of atherosclerotic origin; BMI—body mass index; DBP—diastolic blood pressure; M/F—males/females; SBP—systolic blood pressure.

**Table 3:** Lipid parameters and lipid lowering drugs between 1985 and 2015–2018 in individuals with severe dyslipidemia, Czech Republic.

	1985	1988	1992	1997/98	2000/01	2006–2009	2015–2018	*p* for trend
Males								
Total cholesterol, mmol/L	9.10 ± 1.39	8.88 ± 0.89	8.77 ± 1.41	7.92 ± 0.75	7.87 ± 0.75	7.98 ± 0.93	7.65 ± 0.99	<0.001
HDL-cholesterol, mmol/L	1.24 ± 0.36	1.26 ± 0.39	1.32 ± 0.33	1.22 ± 0.28	1.24 ± 0.31	1.22 ± 0.29	1.19 ± 0.25	ns
Non-HDL-cholesterol; mmol/L	7.85 ± 1.45	7.61 ± 1.02	7.39 ± 1.29	6.62 ± 0.70	6.61 ± 0.76	6.65 ± 0.74	6.46 ± 1.07	<0.001
Triglycerides, mmol/L	-	-	-	3.14 ± 3.29	2.53 ± 2.13	3.23 ± 2.99	2.28 ± 2.67	ns
LDL-cholesterol, mmol/L	-	-	-	5.53 ± 0.48	5.58 ± 0.53	5.56 ± 0.50	5.45 ± 0.41	ns
Lipid lowering drugs (%)	-	-	-	9 (7.3)	13 (8.6)	3 (4.4)	4 (10.5)	ns
Females								
Total cholesterol, mmol/L	8.95 ± 0.89	8.82 ± 0.83	8.78 ± 0.75	8.11 ± 0.82	7.97 ± 0.84	7.93 ± 0.66	7.69 ± 0.48	<0.001
HDL-cholesterol, mmol/L	1.57 ± 0.42	1.57 ± 0.37	1.58 ± 0.41	1.44 ± 0.32	1.44 ± 0.38	1.49 ± 0.37	1.56 ± 0.35	0.011
Non-HDL-cholesterol, mmol/L	7.38 ± 0.90	7.26 ± 0.96	7.20 ± 0.86	6.67 ± 0.85	6.51 ± 0.79	6.44 ± 0.69	6.13 ± 0.43	<0.001
Triglycerides, mmol/L	-	-	-	2.15 ± 1.08	2.06 ± 1.22	1.98 ± 1.09	1.53 ± 0.72	0.012
LDL-cholesterol, mmol/L	-	-	-	5.71 ± 0.72	5.59 ± 0.59	5.55 ± 0.50	5.44 ± 0.33	0.046
Lipid lowering drugs (%)	-	-	-	5 (3.4)	12 (6.9)	3 (4.9)	1 (3.9)	ns

Data are presented as means ± standard deviation or frequency (percentage); ns—non significant, indicating a *p* value > 0.1.

## Data Availability

The data underlying the results presented in this analysis are available at http://www.ftn.cz/data-monica-1117/ (accessed on 1 July 2023).
